# A Quantitative Measurement Method for Nuclear-Pleomorphism Scoring in Breast Cancer

**DOI:** 10.3390/diagnostics14182045

**Published:** 2024-09-14

**Authors:** Chai Ling Teoh, Xiao Jian Tan, Khairul Shakir Ab Rahman, Ikmal Hisyam Bakrin, Kam Meng Goh, Joseph Jiun Wen Siet, Wan Zuki Azman Wan Muhamad

**Affiliations:** 1 Department of Electrical and Electronics Engineering, Faculty of Engineering and Technology, Tunku Abdul Rahman University of Management and Technology (TAR UMT), Jalan Genting Kelang, Setapak, Kuala Lumpur 53300, Malaysia; 2 Biomedical and Bioinformatics Engineering (BBE) Research Group, Centre for Multimodal Signal Processing, Department of Electrical and Electronic Engineering, Faculty of Engineering and Technology, Tunku Abdul Rahman University of Management and Technology (TAR UMT), Jalan Genting Kelang, Setapak, Kuala Lumpur 53300, Malaysia; 3 Sports Engineering Research Centre (SERC), Universiti Malaysia Perlis (UniMAP), Arau 02600, Perlis, Malaysia; 4 Department of Pathology, Hospital Tuanku Fauziah, Jalan Tun Abdul Razak, Kangar 01000, Perlis, Malaysia; 5 Department of Pathology, Faculty of Medicine and Health Sciences, Universiti Putra Malaysia (UPM) Serdang, Serdang 43400, Selangor, Malaysia; 6 Institute of Engineering Mathematics, Universiti Malaysia Perlis (UniMAP), Kampus Pauh Putra, Arau 02600, Perlis, Malaysia; 7 Centre of Excellence for Advanced Computing (ADVCOMP), Universiti Malaysia Perlis (UniMAP), Arau 02600, Perlis, Malaysia

**Keywords:** quantitative measurement, modelling, morphological features, nuclear pleomorphism, nuclear atypia scoring, breast cancer

## Abstract

Background/Objectives: Nuclear pleomorphism, a crucial determinant of breast cancer grading under the Nottingham Histopathology Grading (NHG) system, remains inadequately quantified in the existing literature. Motivated by this gap, our study seeks to investigate and establish correlations among morphological features across various scores of nuclear pleomorphism, as per the NHG system. We aim to quantify nuclear pleomorphism across these scores and validate our proposed measurement method against ground-truth data. Methods: Initially, we deconstruct the descriptions of nuclear pleomorphism into three core elements: size, shape, and appearance. These elements are subsequently mathematically modeled into equations, termed $E_{Size}$, $E_{Shape}$, and $E_{Appearance}$. These equations are then integrated into a unified model termed Harmonic Mean (*HM*). The *HM* equation yields a value approaching 1 for nuclei demonstrating characteristics of score-3 nuclear pleomorphism and near 0 for those exhibiting features of score-1 nuclear pleomorphism. Results: The proposed *HM* model demonstrates promising performance metrics, including Accuracy, Recall, Specificity, Precision, and F1-score, with values of 0.97, 0.96, 0.97, 0.94, and 0.95, respectively. Conclusions: In summary, this study proposes the *HM* equation as a novel feature for the precise quantification of nuclear pleomorphism in breast cancer.

## 1. Introduction

To date, breast cancer remains a global challenge. Recent reports have found that the incidence rates of breast cancer are soaring in developed countries, whereas high mortality rates are reported in lesser-developed countries, mainly due to late diagnoses [1,2,3]. In 2020, a total of 684,996 deaths (15.5% of all cancer cases amongst women) were reported, leading to the disease being proclaimed as the leading cause of mortality (cancer) amongst women across the globe [4,5]. The NHG system is a semi-quantitative system recommended by the World Health Organization (WHO), a modified grading procedure originating from the Scarff–Bloom–Richardson grading system [6]. Nowadays, the NHG system is widely used as the gold standard for breast cancer grading purposes, where nuclear pleomorphism is one of the main features underpinning the final grade of this grading procedure [7].

Breast cancer is a non-communicable disease which emerges in variegated forms, is self-subsistent, and interacts dynamically with its microenvironment through an adaptive process. Given these heterogeneous properties, scoring of nuclear pleomorphism in accordance with the semi-quantitative NHG system could be challenging. Table 1 shows the description of the semi-quantitative NHG system in nuclear pleomorphism, with scores ranging from 1 to 3 [7]. 

## 2. Related Works

Grading of nuclear pleomorphism is tedious, cumbersome, time-consuming, and highly susceptible to variations in the experience levels of histopathologists [8,9]. The conventional grading procedure suffers from inter- and intra-observer variability and could impinge the grading outputs, leading to suboptimal diagnostic and prognostic outcomes. Thanks to the advancements in imaging engineering, the introduction of the whole-slide-imaging scanner has made medical image processing/analysis possible. This has prompted the exploration of repeatable and reproducible algorithms in the detection of nuclear pleomorphism using variegated approaches.

Recent works pertaining to the detection of nuclear pleomorphism can be characterized into two main approaches: handcrafted- and learned-features approaches. Using the handcrafted-features approach, Das, Nair, and Peter proposed a kernel-based Fisher discriminant analysis on the Riemannian manifold (KFDAR), which takes advantage of the kernel trick to embed the non-linear Riemannian manifold M into a higher-dimensional linear Hilbert space H [10]. This subspace was then reduced to a lower-dimensional and more discriminative subspace wherein the samples can be linearly separated. The log-Euclidean metric and the two symmetrized Bregman divergences, the Stein and Jeffrey divergences, are the three Riemannian distance metrics that constitute the basis of the kernel technique developed for the Hilbert space-embedding and kernel-discriminant analysis. The experimental outcomes demonstrated that the proposed mapping to a highly discriminative space had been successful in separating the histopathology images belonging to different cancer grades. As a result, the proposed method outperformed some existing algorithms for cancer grading, both qualitatively and quantitatively.

In the same year, the same team worked on another approach in nuclear-pleomorphism detection via sparse coding and dictionary-learning on symmetric positive definite (SPD) matrices for the grading of breast cancer [11]. The motivation for this method mainly lies within the succession of SPD matrices arising from bundling the difficult approaches within computer vision and machine learning. A convex issue in the higher-dimensional RKHS space was created from the sparse coding issue on the SPD manifold Sn+ in order to better make use of the separability of the cancer grades. The suggested covariance-based SPD matrices were modelled as the sparse merging of Riemannian dictionary atoms, which together constitute a Riemannian manifold. By embedding kernels from the log-Euclidean metric to reproduce the kernel Hilbert space, Jeffrey and Stein divergences, and comparison with the non-kernel-based affine-invariant Riemannian metric, the nonlinearity of the SPD manifold is addressed. The task includes utilizing the kernel approach for the Riemannian manifold’s Hilbert space-embedding, which can improve breast cancer tissue discrimination through the use of sparse representation over learned dictionaries. The performance of the proposed method was found to be better than those of some existing algorithms, in both quantitative and qualitative analysis. The proposed work found that the learned geodesic distances with the Riemannian dictionary atoms were more sensitive and appropriate for the nuclear-pleomorphism score.

In 2020, Das et al. [9] proposed a Riemannian manifold method, a non-Euclidean framework used to investigate the potential for active learning on the nuclear-pleomorphism score. An active learning technique was used in a batch-mode framework that flexibly determined the appropriate batch size as well as the group of samples to query by using the submodular optimization framework. The score of the cancer nuclear pleomorphism depends on the batch-mode active learning, which relies on the Riemannian manifold. This flexibly recognizes the examples used for manual labelling, including the complexities and usefulness of tissue samples as well as the investment cost of labelling data. The Transductive Multi-Label Learning (TRAM) technique was used to replace the need for manual annotation by predicting the class labels of the histological breast carcinoma pictures. Jeffrey divergence, Stein divergence, and log-Euclidean metric were the three kernelized Riemannian metric variations that were subjected to sub-modularity-based dynamic batch-mode active learning and evaluated with contemporary algorithms to define the breast cancer nuclear-pleomorphism grading. Given that it utilizes data from unlabeled samples, adaptive Batch-Mode Active Learning’s results on the Riemannian metric indicates better performance than some existing approaches for evaluating the nuclear atypia in breast cancer.

Salahuddin et al. [12] proposed a novel approach to data sampling that made use of formal concept analysis. The proposed pattern-based hyper-conceptual sampling was a novel mix of conventional sampling methodologies. Formal concepts served as the foundation for the sampling process. The proposed method begins with a conversion to a formal concept analysis and then a conversion is made to an object pattern table. For pattern reduction, the proposed method made use of the hyper-context method. The sample was then generated using the coupling sampling process. To assess the quality of the samples, machine learning experiments are implemented. The performance of the suggested approach was justified by the results of another sampling technique. Using binary distribution, the final sample comprises a very condensed sample that accurately represents the functional dependencies and correlations found in the original dataset. As a result, the proposed method was found promising in producing outcomes that are competitive.

Wan et al. [13] introduced a computer-aided grading system which works dependently with multi-level characteristics and cascaded Support Vector Machine (SVM) classification. To objectively define morphological patterns and understandable concepts from the breast cancer tissue images, pixel-, object-, and semantic-level features were first retrieved. The nuclei from the images were segmented using an enhanced hybrid active contour model-based segmentation technique. In combination with object-level (architecture) features and pixel-level (texture) features, the semantic-level features such as the proportions of nuclei were abstracted with a Convolutional Neural Networks (CNN) approach and defined corresponding to various grade levels. This resulted in an interesting mix of image characteristics that may perform better than either feature subtype on its own. The performance was maximized by utilizing several feature sets collected at various levels; a cascaded technique was used to train multiple SVM classifiers by using the composition of the feature subtypes. To increase the accuracy of the grading process, a cascaded multi-class classification method was used to pyramidally classify images in a series of more-demanding categorization activities. The final class of the cancer grade was calculated by averaging the results of the various SVM classifiers. The proposed method was found computationally effective and adaptable to huge datasets, as the proposed method used only a three-layer CNN model and concurrent processing.

Faridi et al. [14] proposed an automated computer-aided detection (CAD) system that comprises methodologies such as detection, segmentation, and scoring of nuclear pleomorphism. The proposed CAD system starts with the pre-processing stage, such that the hematoxylin and eosin (H/E)-stained input images are first unmixed into their respective sub-component channels. The cores of malignant nuclei were captured in the nuclei-borders analyses that were extracted in Step Two after the preprocessed image had been subjected to morphological procedures and a Difference of Gaussian filter. In the segmentation stage, the segmented nuclei were graded to fulfil a requirement of the NHG system. Finalizing the suggested approach requires scoring segmented nuclei in accordance with the derived features using four criteria that distinguish between malignant and healthy nuclei. Features for nuclear-pleomorphism scoring include the size of the nuclei, the density of the chromatin, the regularity of the contours, and the presence of nucleoli. The proposed CAD system was found promising, and demonstrated an improved accuracy as compared to some existing techniques in the detection of malignant nuclei, with an accuracy of 86.6%. 

In the learned-features approach, recent studies have used the deep CNN architecture for grading breast tumors. For cancer grading purposes in nuclear pleomorphism, Wan et al. proposed mixed CNN-derived semantic-level descriptors together with the object-based data and pixel-based data [13]. Another CNN-based approach was proposed by Mollahosseini and Mahoor, namely, the Deep Belief architecture based Deep Neural Network (DBN-DNN) and Multi-Resolution Convolutional Network (MR-CN) together with Plurality Voting (MR-CN-PV) models, which were built on Restricted Boltzmann Machines (RBM). The proposed method was a multitask CNN architecture that can simultaneously forecast the cancer malignancy attribute and level of magnification.

Numerous variegated forms of the CNN architecture have also been proposed for breast cancer grading focusing on nuclear pleomorphism, including, for example, deep belief networks [15], residual networks [16,17], and inception networks [18,19]. The deep CNN architecture had been used by [19,20] for grading breast tumors. The use of these deep neural networks had demonstrated promising performance in the detection of nuclear pleomorphism. These learning-based algorithms, however, require high-performance computing resources, for example, graphics processing units (GPUs), and were found extremely computationally intensive. 

Das et al. [21] proposed a semi-supervised learning framework focusing on nuclear atypia scoring (NAS) with a deep neural network-based generative adversarial training, namely, NAS-SGAN, with a minimal labelled dataset. The proposed NAS-SGAN model comprises a generator and a discriminator model that has undergone adversarial training with both labelled and unlabeled samples. The discriminator model was created as an unsupervised model that contained layers, as opposed to the supervised model. The proposed model retrieved information pertaining to the population of data by removing discriminative features. The generator model was learned using a consistent attribute corresponding objective function that used compounded GAN architecture. Although a small number of labelled samples were used, experimental research demonstrated that the proposed model could achieve promising accuracy in distinguishing between various cancer grades, enhancing the durability and precision score of the system.

Karimi and Danyali [22] proposed a combination of the CNN for feature extraction and a two-layer Long Short-Term Memory (LSTM) for detection of nuclear pleomorphism in breast cancer. The size of the histopathology images and the limited amount of training data compel the introduction of a patch-based technique. First, the most significant features in the image were identified. Next, a three-hidden-layer CNN was created and used for feature extraction and to categorize the patches separately. For image grading, the LSTM network was used to take into account all features of an image concurrently. The novelty of the proposed method lies within the capability to distinguish between different cancer grades in accordance with the nuclear-pleomorphism scores without performing the nuclei-segmentation stage.

Alom et al. [19] proposed an Inception Recurrent Residual Convolutional Neural Network (IRRCNN) model, comprised of binary and multi-class approaches, for nuclear-pleomorphism scoring purposes. The IRRCNN, recognized as a strong Deep CNN (DCNN) model that integrates the strong performance of the Residual Network (ResNet), Recurrent Convolutional Neural Network (RCNN), and Inception Network (Inception-v4). The proposed IRRCNN was found to outperform the existing methods, including the Inception Networks, Residual Networks, and RCNNs. Testing and validation were performed using two publicly accessible datasets, namely, the Breast Cancer Classification Challenge 2015 and BreakHis. In terms of the patch-based, picture-based, patient-level, and image-level classification, the detection outputs of the proposed IRRCNN were compared with some existing methods using state-of-the-art machine learning and deep learning-based methodologies. The proposed IRRCNN was found promising, achieving higher outputs in terms of area under the curve (AUC) and overall detection accuracy. 

Jiang et al. [20] proposed a CNN-based network, namely, the Breast Cancer Histopathology Image Categorization Network (BHCNet), for the categorization of breast cancer histopathology images. The proposed BHCNet comprises a Squeeze and Excitation (SE) ResNet module which could achieve comparable performance with fewer parameters. The proposed method implemented a new learning-rate scheduler, namely, the Gauss error scheduler, enabling the BHCNet to achieve promising performance without a comprehensive fine-tuning process.

Rakhlin et al. [23] proposed a computational strategy that depends on deep convolution neural networks for the categorization of breast cancer histopathology images. The gradient-boosted trees classifier and numerous deep neural network topologies were used in the proposed detection module. Strong data augmentation and deep convolutional features retrieved at various scales with freely available CNNs trained on ImageNet were used to boost the classifier’s robustness. Furthermore, the proposed module implemented a precise and susceptible-to-overfitting implementation of the gradient boosting algorithm. The proposed module achieved 87.2% accuracy for the four-class categorization task. For a two-class classification task, the proposed module demonstrated 93.8% in accuracy, 97.3% in AUC, and 96.5/ 88.0% in sensitivity/specificity in the detection of breast cancer at the high-sensitivity operational point. 

In both handcrafted- and learned-features approaches, to date, in-depth analysis of the description of nuclear pleomorphism in accordance with the NHG system (as in Table 1) is very limited. Detection or modelling algorithms fully aligned with such descriptions across different scores are not available. There are a number of recent works that demonstrate promising capability in the detection of nuclear pleomorphism in breast cancer. These methods, however, partly or entirely differ from the description stipulated by the NHG system, barricading their implementation in real-world applications. In recent years, quantitative measurement and modelling of nature structures, for example, fetal cardiovascular structures [24], tubule structures [25], and androgenetic alopecia via hair diameter measurement [26], are emerging. These approaches reveal the in-depth features underpinning the object of interest and provide a systematic approach to understanding complex phenomena by capturing the underlying relationships and interactions between different variables [27,28]. As this approach aligns with human perception, trust can thus be established in the output model. Motivated by these determinations, the contributions of the present study are as follows: (1) to investigate and correlate morphological features across different scores concerning nuclear pleomorphism in accordance with the NHG system; (2) to quantify the nuclear pleomorphism via mathematical modelling; and (3) to validate and benchmark the proposed measurement method against the ground truth. The main novelty of the proposed measurements over the other existing findings is that the proposed measurements provide a clear morphological meaning for nuclear pleomorphism across different scores, alongside producing measurable output that enables quantification of qualitative nuclear-pleomorphism features in accordance with the NHG system.

The paper is organized as follows. Section 3 provides a detailed description of the proposed methods. Section 4 describes the dataset and presents the results obtained from this study. The limitations of the study and ideas for future work are given in Section 5. Section 6 summarizes the present study.

## 3. Methods

### 3.1. Theoretical Framework and Mathematical Modelling

**Figure 1:**
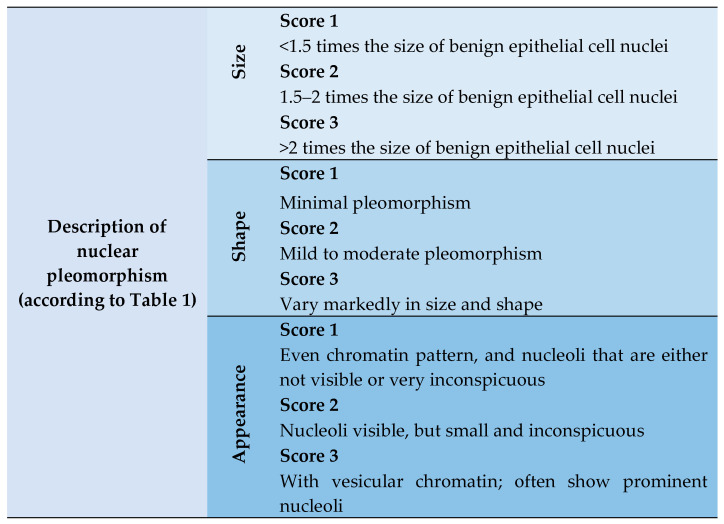
The breakdown of descriptions for nuclear pleomorphism into the three main elements: size, shape, and appearance.

Upon scrutinizing Table 1, the descriptions of nuclear pleomorphism across scores 1 to 3 can be broken down into three main elements: size, shape, and appearance. Figure 1 illustrates the aforementioned breakdown.

A natural question now is how to correlate the three main elements into one usable model to effectively quantify the qualitative descriptions of the nuclear pleomorphism. To achieve this, three mathematical equations are proposed, namely, $E_{Size}$, $E_{Shape}$, and $E_{Appearance}$, for size, shape, and appearance elements, respectively. In this study, an equation, namely, *HM*, is proposed to integrate these three equations into a single, usable model. We hypothesize that the *HM* value for nuclear pleomorphism would fall within the range of [0, 1]. Specifically, the equation expects that a score-3 nuclear pleomorphism has an *HM* value approximated to 1, whereas a score-1 nuclear pleomorphism is approximated to 0.

The mathematical model of the size element is straightforward. The ratio of nucleus size variation is computed as follows:

(1) $$E_{PSize}=\frac{x_{i}-k}{k};\;such\;that\;1\leq i\leq N$$

(2) $$E_{Size}=\left\{\begin{array}{c} 1,\;if\;E_{PSize}\geq 1\\ E_{PSize},\;if\;0.5\leq E_{PSize}<1\\ 0,\;if\;E_{PSize}<0.5 \end{array}\right.$$

where $x_{i}$ denotes the size of the *i*th nucleus; *N* denotes the maximum number of nuclei; and *k* denotes a constant which refers to the size of the benign epithelial cell nuclei. In this study, the *k* constant is set as 849. The proposed *k* constant is determined systematically based on a comprehensive analysis of 200 benign epithelial cell nuclei. The 200 benign epithelial cell nuclei were first segmented using the CellProfiler 3.0 software, using the same procedure/setting as proposed in Step 2 in Section 3.2. Next, the mean value, in pixel area, of the benign epithelial cell nuclei was calculated. This pixel area was then used as the *k* constant herein. Prior to proceeding to the next step, Equations (1) and (2) were tested and validated using an independent dataset with the size of 150 nuclear pleomorphisms (specifically, 50 nuclear pleomorphisms from Scores 1, 2, and 3, respectively), to justify the applicability of the proposed *k* constant. The mathematical model for size element comprises a two-stage calculation, namely, $E_{PSize}$ and $E_{Size}$. The $E_{PSize}$ is first calculated to determine the nucleus size variation, whereas $E_{Size}$ is then used to integrate rules in accordance with the descriptions of nuclear pleomorphism across different scores. The equation yields a value of 1 when the size of the nucleus is >2 times the size of the benign epithelial cell nuclei, whereas a value approaching 0 is returned when the size of the nucleus is 1.5 to 2 times or <1.5 times of the benign epithelial cell nuclei.

To model the shape element, an isoperimetric quotient is used. The equation is as follows:

(3) $$E_{Shape}=1-\frac{4\pi *Ar}{{Peri}^{2}}$$

where *Ar* and *Peri* denote Area and Perimeter, measured in pixel values, respectively. The core idea of Equation (3) is to differentiate between a circular and an irregularly shaped object using a single value within the range of [0, 1]. For a nucleus in a perfect circle shape, the equation yields a value of 0, reflecting the description of score-1 nuclear pleomorphism with an assumption of minimal pleomorphism. For irregular shapes or bizarre objects, the value would be greater than 0 (or approaching 1), reflecting the description of score-3 nuclear pleomorphism, with an assumption of marked variation in shape.

**Figure 2:**
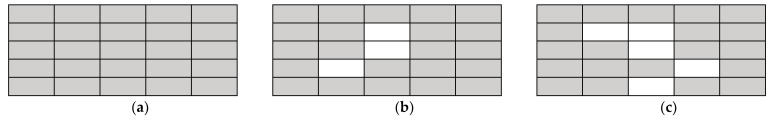
Sample synthetic images with *Hol* of 0, 3, and 5, where grey and white colors denote the foreground and background. (**a**) *Hol* = 0; (**b**) *Hol* = 3; (**c**) *Hol* = 5.

Motivated by one of the previous works, specifically one on the study of nucleus shape in breast histopathology images [8], the appearance element is modelled as follows:

(4) $$E_{Appearance}=\frac{Hol}{Ar}$$

where *Hol* denotes the number of hollow pixels in the cell nucleus (examples in Figure 2), reflecting the pattern and distribution of chromatin and visibility of nucleoli in the cell nucleus. The equation yields a value approaching 1 if and only if the hollow pixels are dominant in the nucleus, whereas a value approaching 0 is returned if and only if the hollow pixels are scarce.

The *HM* equation is then implemented to integrate the three elements (i.e., size, shape, and appearance) into one usable model by integrating Equations (2) to (4) into Equation (5), as follows:

(5) $$HM=\frac{\tau }{w_{1}\frac{1}{E_{Size}}+w_{2}\frac{1}{E_{Shape}}+w_{3}\frac{1}{E_{Appearance}}};$$

$$such\;that\;w_{1}=\left\{\begin{array}{c} 0,\;if\;E_{Size}=0\\ w_{1},\;otherwise \end{array}\right.$$

$$such\;that\;\tau =\left\{\begin{array}{c} 2,\;if\;w_{1}=0\\ 3,\;otherwise \end{array}\right.$$

where $w_{1}$, $w_{2}$, and $w_{3}$ are the weightage of $E_{Size}$, $E_{Shape}$, and $E_{Appearance}$, respectively. It is important to remark that when $E_{Size}$ = 0 (based on Equation (2)), $w_{1}$ is set as 0 (and τ is set as 2, as the *HM* equation now considers only two elements) and rules out the consideration of $E_{Size}$ in the *HM* equation. This is mainly because, mathematically, a division by a zero would result in an infinity value. In this study, all three elements shared same weightage, and thus $w_{1}$, $w_{2}$, and $w_{3}$ are set as 1. The *HM* is defined as the reciprocal of the arithmetic mean of the reciprocals of the three elements in nuclear pleomorphism. This equation provides a method to aggregate the three features, giving equal importance to each element (provided that $w_{1}$, $w_{2}$, and $w_{3}$ are set as 1). The weightage of each element can be adjusted by modifying the value of $w_{1}$, $w_{2}$, and $w_{3}$ and thus provide higher importance to a specific element. The *HM* equation yields a value approaching 1 when the nucleus demonstrates characteristics of score-3 nuclear pleomorphism, whereas a value approaching 0 is returned when the nucleus demonstrates characteristics of score-1 nuclear pleomorphism.

### 3.2. Methodology Pipeline

The implementation of proposed equations concerning the size, shape, and appearance, as well as the *HM* value, in quantifying the nuclear pleomorphism is simple. The methods are as follows:

1. Pre-processing: Extract the H-channel of the input breast histopathology images by converting the RGB input images into optical density space via singular value decomposition [29].
2. Nucleus segmentation: Segment the nucleus using CellProfiler 3.0 [30].
3. Post-processing: If necessary, manual intervention from an expert is involved, such that the cell boundary (pixel-based) is manually edited under the expert’s supervision.
4. Calculation: Calculate the $E_{Size}$, $E_{Shape}$, and $E_{Appearance}$, using the Equations (2) to (4), respectively.
5. Measurement of nuclear pleomorphism: Quantify and measure the nuclear pleomorphism of a nucleus using the *HM* equation (Equation (5)).

Perfect nucleus segmentation is known as a highly challenging task in which continuous efforts are made over the years to improve and close the research gaps [31,32]. It is important to remark that in this study, the main purpose is to quantify the nuclear-pleomorphism scoring in accordance with the NHG system. The features extracted in the subsequent stages however are closely related to the succession of the nucleus segmentation stage. Thus, a semi-supervised nucleus segmentation method is employed such that the Cell Profiler 3.0 [30] is used as a software tool to segment the nuclei from the H-channel of the input histopathology images. To obtain the H-channel, the stain-unmixing method is implemented by converting the RGB input images to optical density space via singular value decomposition [29]. The segmentation outputs are reviewed and monitored by a histopathologist to ensure segmentation is performed accordingly. If necessary, manual intervention is used. This serves as a baseline to justify that the features extracted in the subsequent stages are sound and reflect the characteristics of nuclear pleomorphism across different scores. In Steps 4 and 5, calculation and quantitative measurement of nuclear pleomorphism are performed by using the proposed equations, namely, $E_{Size}$, $E_{Shape}$, $E_{Appearance}$, and *HM*. 

To validate the hypothesis and the applicability of the proposed measurement method, the measurement outputs are tested and validated against the ground truth provided by the histopathologist. For this purpose, a baseline classifier is used, namely, SVM [33,34]. The classification was performed using a fivefold cross-validation SVM classifier with the Radial Basis Function (RBF) kernel. The data are randomly divided into five equal pieces. Each selected piece is chosen as a test set, with training performed on the remaining portion of the data. The cross-validation is then repeated five times, with each subsample used exactly once as the validation data. All the observations are used for both training and validation, and each observation is specifically used for validation.

Evaluation metrics, for example, Accuracy, Recall, Specificity, Precision, and F1-score, are used to statistically analyze the performance against the ground truth. Table 2 summarizes the evaluation metrics used in this study; the tubule that was correctly labeled as a tubule is referred to as a True Positive (*TP*), the non-tubule that was correctly labeled as a non-tubule is referred to as a True Negative (*TN*), the non-tubule that was wrongly labeled as a tubule is referred to as a False Positive (*FP*), and the tubule that was wrongly labeled as a non-tubule is referred to as a False Negative (*FN*). A promising method would demonstrate high values in all the evaluation metrics.

## 4. Dataset, Results, and Discussions

### 4.1. Dataset

The dataset used in this study comprises real histopathology slides of breast cancer which were locally collected in Malaysia. The breast histopathology slides were obtained from the Pathology Department, Hospital Tuanku Fauziah, Kangar, Perlis, Malaysia. A total of 48 breast histopathology slides from 48 patients (one slide per patient) with anonymized identities were collected retrospectively. These slides were prepared under a standard staining procedure using the hematoxylin and eosin (H/E) stains. The staining procedure is aligned with the protocols of the Ministry of Health, Malaysia. An Aperio CS2 whole-slide-imaging scanner was used to convert the histopathology slides into digital form. The scanning procedure was performed under the careful supervision of an experienced histopathologist and an instrument specialist to ensure the histopathology slides were scanned under fixed instrument parameters (e.g., input format and objective lenses). The ethical protocol of this study has been approved by the Medical Research and Committee of the National Medical Research Register (NMRR) Malaysia, with protocol number: NMRR-21-949-58903. For validation and benchmarking purposes, a total of 600 nuclei were demarcated by a histopathologist from the 48 breast histopathology slides, such that 200 nuclei from each score were obtained via convenient sampling. The 600 demarcated nuclei served as ground truth in this study. 

### 4.2. Analysis on the Outputs of $E_{Size}$, $E_{Shape}$, $E_{Appearance}$, and HM

**Figure 3:**
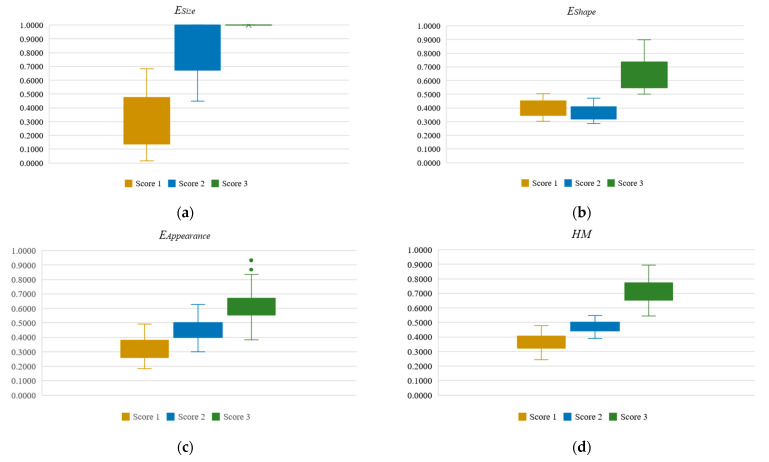
The boxplots of $E_{Size}$, $E_{Shape}$, $E_{Appearance}$, and *HM* for scores 1 to 3 for nuclear pleomorphism using 300 ground truth, such that 100 nuclei were included for each score. (**a**) Analysis for $E_{Size}$; (**b**) Analysis for $E_{Shape}$; (**c**) Analysis for $E_{Appearance}$; (**d**) Analysis for *HM*.

Figure 3 shows the boxplots analysis of $E_{Size}$, $E_{Shape}$, $E_{Appearance}$, and *HM* for scores 1 to 3 for nuclear pleomorphism using 300 ground truth, such that 100 nuclei were included for each score in the analysis. Based on Figure 3a, it is evident that the $E_{Size}$ values of score-1 and score-2 nuclear pleomorphism exhibit clear separation, whereas the $E_{Size}$ values for scores 2 and 3 are overlapping. Notably, the $E_{Size}$ of score-3 nuclear pleomorphism remains consistent across all 100 nuclei, indicating remarkable variation in nucleus size (i.e., >2.0 times). 

In Figure 3b, score-1 and score-2 nuclear pleomorphism display overlapping data in $E_{Shape}$, with the $E_{Shape}$ outputs from score-2 nuclear pleomorphism slightly lower than those of score-1. Conversely, score-3 nuclear pleomorphism exhibits distinct separation from the others. This delineation is justifiable, as marked variation in shape is a common feature of score-3 nuclear pleomorphism. In the context of breast cancer, pleomorphism refers to variations in the size, shape, and structure of nucleus cells, particularly when compared to benign pre-existing epithelial cells [7]. In this study, $E_{Size}$ and $E_{Shape}$ are proposed primarily to identify variations in size and shape, respectively. As variation in size and shape are key features of pleomorphism, the proposed $E_{Size}$ and $E_{Shape}$ complement each other to effectively capture pleomorphism across scores 1 to 3. Therefore, we infer that the score-2 nucleus pleomorphism sampled herein demonstrates variation in size rather than shape, resulting in a boxplot with lower values compared to score-1 nuclear pleomorphism, as depicted in Figure 3b.

In Figure 3c, the outputs of $E_{Appearance}$ across scores 1 to 3 for nuclear pleomorphism comply to the hypothesis made, such that $E_{Appearance}$ yields a value approaching 1 if the hollow pixels are dominant in the nucleus, whereas a value approaching 0 is returned if the hollow pixels are scarce. We could deduce that the *Hol* is sufficiently effective to reflect chromatin pattern and visibility of nucleoli in a nucleus.

Figure 3d shows the boxplots of *HM* analysis across scores 1 to 3 for nuclear pleomorphism. It is evident that the boxplots exhibit clear separation across different scores and the compact boxplots reflect consistency and strong agreement when measuring nuclear pleomorphism using the proposed *HM*, and considering the three main elements, size, shape, and appearance, namely, $E_{Size}$, $E_{Shape}$, and $E_{Appearance}$, respectively.

### 4.3. Measurement Outputs

To further illustrate the applicability of the proposed measurement, three samples from each score are provided in Table 3, detailing the original input nucleus patch, segmentation outputs, and measurement outputs of $E_{Size}$, $E_{Shape}$, $E_{Appearance}$, and *HM*. Based on Table 3, the proposed measurements, $E_{Size}$, $E_{Shape}$, $E_{Appearance}$, and *HM*, appear to be aligned with the hypothesis made in this study, such that the *HM* values for scores 1 and 3 nuclear pleomorphism are approaching 0 and 1, respectively. The *HM* values obtained for the score-1 nucleus are 0.3244, 0.3759, and 0.4382; for score-2, the values are: 0.4816, 0.4997, and 0.4985; and for score-3, the values are: 0.7542, 0.6524, and 0.8404.

### 4.4. Classification Outputs

**Figure 4:**
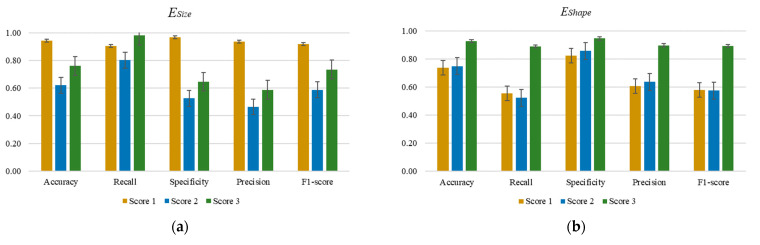

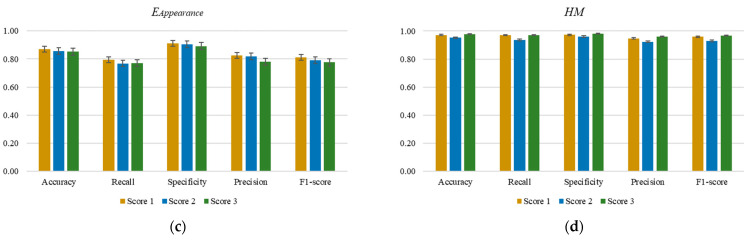
Classification outputs for $E_{Size}$, $E_{Shape}$, $E_{Appearance}$, and *HM*, using the dataset. (**a**) Using the $E_{Size}$ as input feature; (**b**) Using the $E_{Shape}$ as input feature; (**c**) Using the $E_{Appearance}$ as input feature; (**d**) Using the *HM* as input feature.

As detailed in Section 3.2, the SVM with RBF kernel is used and serves as a baseline classifier to assess the viability of the proposed measurement. Here, the proposed $E_{Size}$, $E_{Shape}$, $E_{Appearance}$, and *HM* are used as new input features to measure and discriminate nuclear pleomorphism across different scores. As fivefold cross-validation is used, the mean values of the classification outputs are initially computed. Figure 4 shows the classification outputs for $E_{Size}$, $E_{Shape}$, $E_{Appearance}$, and *HM*, using the dataset. 

Based on Figure 4a–c, using the proposed $E_{Size}$, $E_{Shape}$, and $E_{Appearance}$ alone as an input feature into the classifier is insufficient to discriminate nuclear pleomorphism into different scores accurately. The obtained Accuracy, Recall, Specificity, Precision, and F1-score for scores 1 to 3 are found to be lower than 0.80, denoting sub-optimal classification outcomes. In Figure 4a, the Accuracy, Specificity, Precision, and F1-score obtained for scores 2 and 3 nuclear pleomorphism are relatively low (i.e., <0.80). This suggests that using $E_{Size}$ alone is insignificant to discriminate between score-2 and score-3 nucleus pleomorphism but effective in identifying score-1 nuclear pleomorphism. This is justifiable, as the sizes of the nucleus cell for scores 2 and 3 are overlapping, as shown in Figure 3a. Supplementary features are required to better partition the data into correct categories. In Figure 4b, all evaluation metrics for score-3 nuclear pleomorphism show promising outcomes (i.e., >0.80), but relatively poor outputs are found in Accuracy, Recall, Precision, and F1-score (i.e., <0.80) for both score-1 and score-2 nuclear pleomorphism. This is primarily due to heterogeneous properties of pleomorphism concerning both size and shape features and the finding herein is aligned with the boxplot analysis as in Figure 3b. In Figure 4c, using $E_{Appearance}$ alone as an input feature to the classifier produced slightly better outcomes, compared to the $E_{Size}$ and $E_{Shape}$, with values approaching 0.80, if not better. The classification outcomes, however, are not optimal. Based on Figure 4d, it is evident that the overall performance of the classification outputs is promising, achieving values of more than 0.90 in all evaluation metrics, such that overall values in Accuracy, Recall, Specificity, Precision, and F1-score are 0.97, 0.96, 0.97, 0.94, and 0.95, respectively. The proposed *HM* formulation aligns with the description of nuclear pleomorphism outlined in the NHG system, effectively incorporating all three main elements: size, shape, and appearance. Each element functions as a supplementary feature in the equation, contributing to the delineation and enhancement of classification outcomes. This comprehensive approach enables accurate discrimination of nuclear pleomorphism into different scores. Notably, the error bars in Figure 4d for all evaluation metrics are relatively narrow, indicating a strong consensus in classification outcomes with minimal standard deviation.

### 4.5. Ablation Study

To further justify the superiority of the proposed *HM* equation against the proposed equations of $E_{Size}$, $E_{Shape}$, and $E_{Appearance}$, an ablation study is conducted. Here, specific input features are systematically removed from consideration while keeping the classifier parameters unchanged. This approach allows for the evaluation of the individual contributions of each feature to the overall performance. By systematically eliminating different input features and observing the resulting changes, insights into the importance and effectiveness of various features can be gleaned. Table 4 presents the F1-scores obtained from the ablation study, indicating the performance with different features used as input to the classifier. Based on Table 4, it is evident that the F1-scores for HM are highest across scores 1 to 3, with an overall output of 0.95.

### 4.6. Benchmarking with Existing Quantitative Features

**Figure 5:**
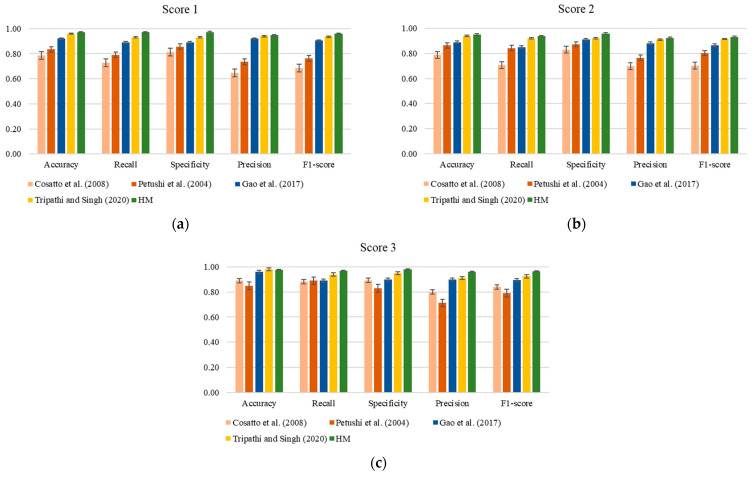
Classification outputs comparing the existing quantitative features and deep learning methods with the proposed *HM* [35,36,37,38]. (**a**) Score 1; (**b**) Score 2; (**c**) Score 3.

To date, existing quantitative features focusing on nuclear pleomorphism are very limited. Due to the heterogeneous properties of pleomorphism in breast cancer, conventional features, for example, shape, texture, and fitness of the outline [35], as well as morphological and textural features [36], are commonly used. Additionally, to further validate the applicability of the proposed measurement method, recent deep learning methods, for example, the LeNet-5 [37] and CNN [38] methods, were compared with the proposed *HM*. Figure 5 illustrates the classification outputs of the existing quantitative features juxtaposed with the proposed *HM*. From the figure, it is evident that the outputs for all evaluation metrics obtained from the proposed *HM* are consistently higher than those of the outputs obtained from the existing quantitative features [35,36]. Compared with the recent deep learning methods [37,38], the proposed *HM* demonstrates comparable if not better performance. This demonstrates the robustness of the proposed *HM* in discriminating among nuclear pleomorphism scores 1 to 3. The proposed *HM* presents itself as a promising new feature for measuring nuclear pleomorphism in breast cancer.

## 5. Limitations and Future Works

Manual annotation and dataset preparation are known to be expensive, tedious, and time-consuming processes [39,40]. In this study, we conducted an experiment to quantify nuclear pleomorphism by thoroughly investigating the features associated with nuclear pleomorphism as described by the NHG system. The obtained outputs validate the applicability and robustness of the proposed *HM* as a new feature for measuring and discriminating nuclear pleomorphism using different scores. Compared to the crowdsourcing-based datasets, for example (the NuCLS dataset [41]), the dataset used in this study is seen to be small. Thus, we aim to expand and validate our findings using crowdsourcing-based datasets in the future. Furthermore, we plan to develop a fusion-oriented deep learning model to automate the nucleus segmentation stage and utilize the proposed *HM* as an input for measuring nuclear pleomorphism in breast cancer.

## 6. Conclusions

In this study, an in-depth investigation of the nuclei across score-1 to score-3 nuclear pleomorphism is first performed. The insights gained serve as the foundation for the proposed quantitative equations, which detail the three core elements of nuclear pleomorphism, namely, size, shape, and appearance, denoted as $E_{Size}$, $E_{Shape}$, and $E_{Appearance}$, respectively. These equations are then integrated into a single usable model, termed *HM*. The output of the proposed *HM* is a quantitative value falling within the range of [0, 1], for which it is hypothesized that a score-3 nuclear pleomorphism has an *HM* value approximated to 1, while a score-1 nuclear pleomorphism is approximated to 0. To validate the applicability of the proposed measurement, a baseline classifier, namely, the SVM with RBF kernel, is employed. The proposed *HM* demonstrates promising outputs, achieving Accuracy, Recall, Specificity, Precision, and F1-score values of 0.97, 0.96, 0.97, 0.94, and 0.95, respectively. Compared to some existing quantitative features, the proposed *HM* outperforms these features, leading to the conclusion that it could serve as a new feature for measuring nuclear pleomorphism in breast cancer. Additionally, the proposed HM provides a clear morphological meaning for nuclear pleomorphism across different scores, producing measurable output that could be used as a second opinion in standard nuclear-pleomorphism scoring procedures in breast cancer.

## Figures and Tables

**Table 1:** Description of nuclear pleomorphism in the semi-quantitative NHG system.

Description of Nuclear Pleomorphism	Scores
Score-1 nuclei are very similar in size to the nuclei of benign pre-existing epithelial cells (<1.5 times the size), and they show minimal pleomorphism, and an even chromatin pattern, as well as nucleoli that are either not visible or very inconspicuous.	1
Score-2 nuclei are larger (1.5–2 times the size of benign epithelial cell nuclei), with mild to moderate pleomorphism and visible, but small and inconspicuous, nucleoli.	2
Score-3 nuclei are even larger (>2 times the size of benign epithelial cell nuclei), with vesicular chromatin; they vary markedly in size and shape and often show prominent nucleoli.	3

**Table 2:** Evaluation metrics.

Metrics	Equations
Accuracy	$\frac{TP+TN}{TP+TN+FP+FN}$
Recall	$\frac{TP}{FN+TP}$
Specificity	$\frac{TN}{FP+TN}$
Precision	$\frac{TP}{FP+TP}$
F1-score	$\frac{2*Recall*Precision}{Recall+Precision}$

**Table 3:** Sample measurement outputs.

Nuclear Pleomorphism		$E_{Size}$	$E_{Shape}$	$E_{Appearance}$	*HM*
Original Input	Segmentation Outputs *				
**Score 1**					
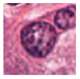	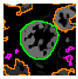	0.3993	0.3661	0.2912	0.3244
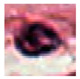	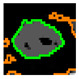	0.5034	0.3407	0.3270	0.3759
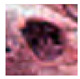	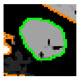	0.0863	0.4715	0.4093	0.4382
**Score 2**					
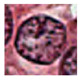	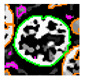	0.7694	0.3861	0.4274	0.4816
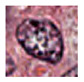	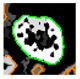	0.5669	0.4360	0.5139	0.4997
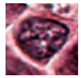	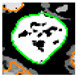	0.6449	0.4153	0.4855	0.4985
**Score 3**					
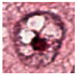	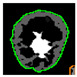	1.0000	0.6479	0.6973	0.7542
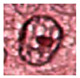	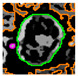	1.0000	0.5269	0.5881	0.6524
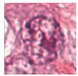	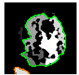	1.0000	0.7049	0.8687	0.8404

* The green color lines show the boundary of the segmented cell nucleus via the CellProfiler 3.0 [30].

**Table 4:** Ablation study.

Feature(s) Included	F1-Score			
	Score 1	Score 2	Score 3	Overall
$E_{Size}$	0.92	0.59	0.74	0.75
$E_{Shape}$	0.58	0.57	0.89	0.68
$E_{Appearance}$	0.81	0.79	0.78	0.79
$E_{Size}$ + $E_{Shape}$	0.67	0.51	0.70	0.63
$E_{Size}$ + $E_{Appearance}$	0.83	0.64	0.71	0.73
$E_{Shape}$+$E_{Appearance}$	0.66	0.61	0.81	0.69
*HM* ($E_{Size}$ + $E_{Shape}$ + $E_{Appearance}$)	**0.96**	**0.93**	**0.97**	**0.95**

Bold fonts highlight the highest output in each column.

## Data Availability

Restrictions apply to the datasets: The datasets presented in this article are not readily available because the data are part of the ongoing study and associated with privacy restrictions. Requests to access the datasets should be directed to the corresponding author.
